# A closed-loop ventilation mode that targets the lowest work and force of breathing reduces the transpulmonary driving pressure in patients with moderate-to-severe ARDS

**DOI:** 10.1186/s40635-023-00527-1

**Published:** 2023-07-14

**Authors:** Laura A. Buiteman-Kruizinga, David M. P. van Meenen, Lieuwe D. J. Bos, Pim L. J. van der Heiden, Frederique Paulus, Marcus J. Schultz

**Affiliations:** 1 Department of Intensive Care, Reinier de Graaf Hospital, Delft, The Netherlands; 2 Department of Intensive Care, Amsterdam University Medical Centers, Location ‘AMC’, Amsterdam, The Netherlands; 3 Department of Anesthesia, Amsterdam University Medical Centers, Location ‘AMC’, Amsterdam, The Netherlands; 4 Department of Respiratory Medicine, Amsterdam University Medical Centers, Location ‘AMC’, Amsterdam, The Netherlands; 5 ACHIEVE, Centre of Applied Research, Faculty of Health, Amsterdam University of Applied Sciences, Amsterdam, The Netherlands; 6 Mahidol-Oxford Tropical Medicine Research Unit (MORU), Mahidol University, Bangkok, Thailand; 7 Nuffield Department of Medicine, University of Oxford, Oxford, UK

**Keywords:** ARDS, Mechanical ventilation, Invasive ventilation, Automated ventilation, Closed-loop ventilation, Driving pressure, Transpulmonary pressure, Mechanical power, Intensity of ventilation

## Abstract

**Introduction:**

The driving pressure (Δ*P*) has an independent association with outcome in patients with acute respiratory distress syndrome (ARDS). INTELLiVENT-Adaptive Support Ventilation (ASV) is a closed-loop mode of ventilation that targets the lowest work and force of breathing.

**Aim:**

To compare transpulmonary and respiratory system Δ*P* between closed-loop ventilation and conventional pressure controlled ventilation in patients with moderate-to-severe ARDS.

**Methods:**

Single-center randomized cross-over clinical trial in patients in the early phase of ARDS. Patients were randomly assigned to start with a 4-h period of closed-loop ventilation or conventional ventilation, after which the alternate ventilation mode was selected. The primary outcome was the transpulmonary Δ*P*; secondary outcomes included respiratory system Δ*P*, and other key parameters of ventilation.

**Results:**

Thirteen patients were included, and all had fully analyzable data sets. Compared to conventional ventilation, with closed-loop ventilation the median transpulmonary Δ*P* with was lower (7.0 [5.0–10.0] vs. 10.0 [8.0–11.0] cmH_2_O, mean difference − 2.5 [95% CI − 2.6 to − 2.1] cmH_2_O; *P* = 0.0001). Inspiratory transpulmonary pressure and the respiratory rate were also lower. Tidal volume, however, was higher with closed-loop ventilation, but stayed below generally accepted safety cutoffs in the majority of patients.

**Conclusions:**

In this small physiological study, when compared to conventional pressure controlled ventilation INTELLiVENT-ASV reduced the transpulmonary Δ*P* in patients in the early phase of moderate-to-severe ARDS. This closed-loop ventilation mode also led to a lower inspiratory transpulmonary pressure and a lower respiratory rate, thereby reducing the intensity of ventilation.

*Trial registration* Clinicaltrials.gov, NCT03211494, July 7, 2017. https://clinicaltrials.gov/ct2/show/NCT03211494?term=airdrop&draw=2&rank=1.

**Supplementary Information:**

The online version contains supplementary material available at 10.1186/s40635-023-00527-1.

## Background

The driving pressure (Δ*P*), an easy to calculate ventilation parameter [1, 2], represents the strain applied to the lung with each breath during invasive ventilation [3]. The Δ*P* has an independent association with outcome in critically ill invasively ventilated patients, including in patients with acute respiratory distress syndrome (ARDS) [4–6]. It has been proposed to adjust ventilator settings so that the Δ*P* becomes or remains low in patients with ARDS, using 15 cmH_2_O as a safe cutoff [1, 7].

Closed-loop ventilation modes are increasingly available for use in critically ill invasively ventilated patients [8]. INTELLiVENT-Adaptive Support Ventilation (ASV) is one sophisticated form of automated, or closed-loop ventilation, wherein tidal volume (*V*_T_), respiratory rate (RR), positive end-expiratory pressure (PEEP) and the fraction of inspired oxygen (FiO_2_) are automatically set and adjusted by a series of algorithms within the ventilator that target a lower work of breathing and a lower force of breathing [9, 10]. INTELLiVENT-ASV then acts within ranges for the end-tidal CO_2_ and the SpO_2_, and limits for maximum airway pressure and PEEP, set by the ICU nurse or doctor. Previous studies of this closed-loop ventilation mode have shown a reduction in Δ*P* with its use, but studies so far included mixed patient groups [11], or exclusively included patients with coronavirus disease 2019 (COVID-19) ARDS [12, 13]. Also, none of these studies used an esophagus balloon catheter for proper measurements of transpulmonary pressures. Indeed, all these studies reported the effect of closed-loop ventilation on the Δ*P* of the respiratory system and not its effects on the transpulmonary Δ*P* [14].

We aimed to determine the effects of INTELLiVENT-ASV on transpulmonary Δ*P* and other ventilation parameters in patients in the early phase of moderate-to-severe ARDS. For this, we designed and conducted a cross-over study, named ‘Does Automated closed-loop ventilation Reduce the DRiving Pressure levels in patients with ARDS (AiRDRoP)’. We hypothesized that the closed-loop ventilation of interest would reduce the transpulmonary Δ*P*.

## Methods

### Study design

This was an investigator-initiated, single-center, randomized cross-over clinical trial conducted at the intensive care unit (ICU) of the Amsterdam University Medical Centers, ‘location AMC’, in Amsterdam, the Netherlands. The study protocol was approved by the local Institutional Review Board (April 13, 2017; 2016_349#B2017211). The study protocol was registered at clinicaltrials.gov (study identifier NCT03211494). Written informed consent was obtained from a legal representative of the patient before inclusion and randomization. A statistical analysis plan was written and finalized before cleaning and closing of the database.

This study was originally designed to have two phases, one randomized cross-over phase, followed by a randomized parallel phase. We prematurely stopped the study because of a sharp increase in use of extracorporeal life support (ELS) in patients with ARDS as part of change in the standard of care at the study site. This meant that it was no longer guaranteed that patients would not receive ELS, i.e., in the second part of the study. Consequently, we stopped inclusions of patients, as use of ELS was an exclusion criterion for this study. We also noticed that in many patients the esophagus balloon catheter was removed after the cross-over phase, because patients became active and doctors saw no need in keeping it in place.

### Patients

Patents were eligible for participation in AiRDRoP if: (1) aged > 18 years; (2) having moderate-to-severe ARDS, according to the current definition for ARDS [15]. Patients were excluded if they were after 24 h following the initial diagnosis of ARDS, and in case of a contraindication for placing an esophagus balloon catheter. We also excluded pregnant patients, terminally ill patients, patients with increased or uncontrollable intracranial pressure, patients receiving therapies that could influence ventilator settings and parameters, and patients previously included in this study.

### Randomization and masking

Patients were randomly assigned in a 1:1 ratio to start with closed-loop ventilation or conventional ventilation for 4 h, after which each patient received ventilation using the alternative ventilation mode. A dedicated, password protected, web-based randomization system (SSL-encrypted website, Sealed Envelope™, London, United Kingdom) was used for non-stratified block randomization using block sizes of 4 patients. Doctors and nurses taking care of the patients could not be blinded because of the nature of the intervention. The investigators analyzing the data, however, remained blinded for the allocated ventilation mode at all times.

### Study interventions

Patients were sedated and if necessary paralyzed according to the local guidelines for analgo-sedation. All patients were to be without spontaneous breathing activity. To guarantee this, an experienced researcher checked the ventilator waveforms and compared set RR with measured RR at each time point data were to be collected. Patients were hemodynamically stabilized before start of the study, meaning that they had received intravenous fluids and if necessary norepinephrine or dobutamine, according to the local protocol.

The same type of ventilator (Hamilton Medical AG, Bonaduz, Switzerland), was used for all patients. All doctors and nurses within the department were extensively trained in use and qualified and experienced with this ventilator, and also the two ventilation modes that were compared.

An esophageal balloon catheter (Cooper Surgical, Trumbull, CT) was inserted, and correct position was confirmed with an occlusion test, as previously described [16]. The catheter was used for collection of pressure data during the cross-over phase of the study, but these data were not disclosed to the bedside doctors or nurses. In other words, they could not be used to adjust ventilator settings.

At initiation of invasive ventilation, the attending doctor or nurse set the ventilator according to the local ventilation protocol that dictates the use of lung-protective ventilator settings with conventional pressure controlled ventilation. Herein, ventilation should use a low *V*_T_ of 6–8 mL/kg predicted body weight (PBW) with a maximum airway pressure limit of 30 cmH_2_O, and PEEP according to the lower PEEP/FiO_2_ table [17]. The lowest PEEP allowed was 5 cmH_2_O. FiO_2_ was adjusted to maintain the peripheral oxygen saturation (SpO_2_) between 92 and 96%. The respiratory rate was adjusted to maintain end-tidal CO_2_ (etCO_2_) to have an arterial pH between 7.25 and 7.45.

At start of closed-loop ventilation, the attending doctor or nurse set the peripheral pulse oximetry (SpO_2_) and end-tidal CO_2_ (etCO_2_) ranges using the same goals as with conventional ventilation. The closed-loop ventilation mode then automatically adjusted *V*_T_, RR, PEEP and FiO_2_ according to a series of software algorithms that continuously target a low work of breathing and a low force of breathing, as previously described [9, 10]. With closed-loop ventilation, the PEEP window was set at 5 to 15 cmH_2_O with a maximum airway pressure limit of 30 cmH_2_O. With start of conventional ventilation, the attending doctor or nurse set the ventilator as described at initiation of invasive ventilation.

### Data collection

Ventilation parameters were collected at the bedside at 32 consecutive time points, 16 time points per each ventilation mode. Every 15 min, at all time points, inspiratory holds and expiratory holds were performed to measure the static ventilation pressures. We collected end-inspiratory airway pressure (Pplat, cmH_2_O), end-inspiratory esophageal pressure (cmH_2_O, inspiratory Pes), end-expiratory airway pressure (PEEP, cmH_2_O), and end-expiratory esophageal pressure (cmH_2_O, expiratory Pes). We also collected measured and set respiratory rate (RR, breaths per minute), tidal volume (*V*_T,_ mL), fraction of inspired oxygen (FiO_2_), end-tidal carbon dioxide (etCO_2_, kPa) and pulse oximetry (SpO_2_, %). In addition, an arterial blood gas was performed 30 min before the end of the block, according to the study protocol.

### Outcomes

The primary outcome was the transpulmonary Δ*P* (Δ*P*_TP_). Secondary outcomes included *V*_T_, respiratory system Δ*P* (Δ*P*_RS_), respiratory system compliance (*C*_RS_), inspiratory transpulmonary pressure (*P*_TP_), PEEP, Pplat and RR.

### Calculations

The following equations were used [1, 18–20]:

1 $$V_{{\text{T}}} \left( {{\text{mL}}/{\text{kg}}\;{\text{PBW}}} \right) = V_{{\text{T}}} /{\text{PBW}};$$

2 $$C_{{{\text{RS}}}} \left( {{\text{mL}}/{\text{cmH}}_{2} {\text{O}}} \right) = V_{{\text{T}}} /\left( {{\text{Pplat}}{-}{\text{PEEP}}} \right);$$

3 $$\Delta P_{{{\text{RS}}}} \left( {{\text{cmH}}_{2} {\text{O}}} \right) = {\text{Pplat}}{-}{\text{total}}\;{\text{PEEP;}}$$

4 $$\Delta P_{{{\text{ES}}}} \left( {{\text{cmH}}_{2} {\text{O}}} \right) = {\text{inspiratory}}\;{\text{Pes}}{-}{\text{expiratory}}\;{\text{Pes;}}$$

5 $$\Delta P_{{{\text{TP}}}} \left( {{\text{cmH}}_{2} {\text{O}}} \right) = \Delta P_{{{\text{RS}}}} {-}\Delta P_{{{\text{ES}}}} ;\;{\text{and}}$$

6 $$P_{{{\text{TP}}}} \left( {{\text{cmH}}_{2} {\text{O}}} \right) = {\text{Pplat}}{-}\Delta P_{{{\text{ES}}}} .$$

### Sample size calculation

We based the power calculation for the randomized cross-over phase of the study on unpublished data from a published cohort of ARDS patients [21], and data from one presented scientific abstract [22]. The power calculation showed that 12 patients would be needed to have 80% statistical power to detect a difference in the Δ*P*_TP_, assuming an effect size (*f*) of 0.25. This number was reached at the moment the study was primarily stopped.

### Statistical analysis

Data are expressed in numbers and proportions for categorical variables and medians [with interquartile ranges] or means (with standard deviations) for continuous variables, where appropriate. Proportions are compared using Chi-squared test or Fisher exact as required by variable distribution; continuous variables are compared using paired *T*-test or Wilcoxon signed-rank where appropriate. Effects are presented with a 95% confidence interval (95% CI).

A repeated measure analysis of variance (ANOVA) was performed to evaluate the effect of ventilation mode over time, to account for the repeated measurements and the time exposure, on Δ*P*_TP_, Δ*P*_RS_ and the other collected ventilation parameters. We performed pairwise comparisons to evaluate the effect of ventilation mode at the individual time points.

Cumulative distribution plots, boxplots, scatterplots and line plots were constructed to visualize Δ*P*_TP_, Δ*P*_RS_ and other ventilation parameters with closed-loop ventilation versus conventional ventilation. In the cumulative distribution plots, vertical dotted lines represent the median of the corresponding value with conventional ventilation, and horizontal dotted lines show the respective proportion of patients reaching each cutoff. In addition, the relationship between ventilation parameters was visualized in plots using least squares method regression.

We performed two post hoc analyses, one wherein we compared respiratory system mechanical power (MP_RS_) and transpulmonary MP (MP_TP_) with closed-loop ventilation to conventional ventilation. We calculated MP_RS_ [23], MP_TP_ [24, 25] and lung elastance (*E*_L_) [24] as follows:

7 $${\text{MP}}_{{{\text{RS}}}} \left( {{\text{J}}/{\text{min}}} \right) = 0.098*{\text{RR}}*V_{{\text{T}}} *\left( {{\text{Ppeak}}{-}\raise.5ex\hbox{$\scriptstyle 1$}\kern-.1em/ \kern-.15em\lower.25ex\hbox{$\scriptstyle 2$} *\Delta P} \right);$$

8 $${\text{MP}}_{{{\text{TP}}}} \left( {{\text{J}}/{\text{min}}} \right) = 0.098*{\text{RR}}*\left( {V_{{\text{T}}}^{2} *\raise.5ex\hbox{$\scriptstyle 1$}\kern-.1em/ \kern-.15em\lower.25ex\hbox{$\scriptstyle 2$} *E_{{\text{L}}} + V_{{\text{E}}} *{\text{PEEP}}} \right);$$

9 $$E_{{\text{L}}} \left( {{\text{cmH}}_{2} {\text{O}}/{\text{L}}} \right) = \Delta P_{{{\text{TP}}}} /V_{{\text{T}}} .$$

In the second post hoc analysis, we used a generalized linear mixed model analysis to improve the inclusion of the effect of time on the ventilation parameters in the analysis, with ventilation mode and time as fixed effects, and patients as random effect.

For the pairwise comparisons, an adjusted *P* value was calculated using Bonferroni method, and a *P* < 0.003 was considered significant. A *P* < 0.05 was considered significant for the other analyses. Missing data were < 1% and imputed with multivariate imputation via chained equations (MICE) by means of predictive mean matching method [26].

All analyses were performed in R version 4.0.3 (R Foundation, Vienna, Austria).

## Results

### Patients

**Fig. 1:**
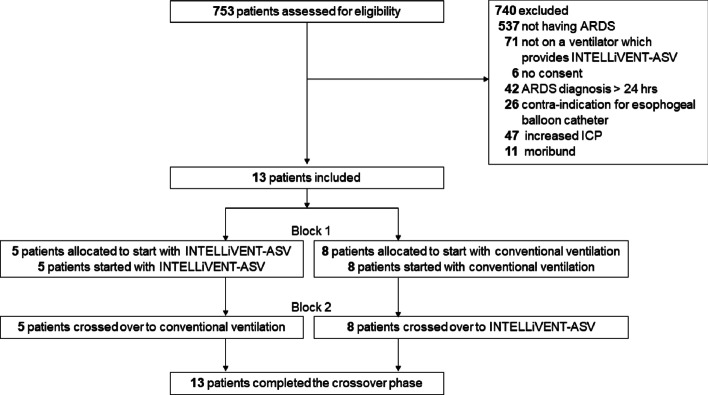
Flow of patients in the study

Between November 3, 2017 and March 1, 2019, 13 patients were included (Fig. 1). The majority of patients were male (62%), the main cause for ARDS was sepsis (Table 1). All patients completed the cross-over phases of the study and were ventilated and switched according to the randomization arm. There were no protocol violations, meaning that patients were ventilated according to randomization at all time points. 5 patients started with closed-loop ventilation, 8 patients started with conventional ventilation.

**Table 1:** Baseline characteristics

	*N* = 13
Gender, male	8 (62)
Age, years	64 (61–71)
Height, cm	177 (174–186)
Weight, kg	84 (74–95)
BMI, kg/m^2^	27 (24–28)
Reason for ICU admission, *n* (%)	
Medical	9 (70)
Surgical	4 (30)
Reason for ARDS, *n* (%)	
Sepsis	8 (62)
Pneumonia	2 (15)
Trauma	3 (23)
APACHE II score	28 (24–31)
ARDS severity, *n* (%)	
Moderate	1 (8)
Severe	12 (92)

Data are median (IQR) or *N*/total (%)

*BMI* body mass index, *ARDS* acute respiratory distress syndrome, *APACHE* Acute Physiology and Chronic Health Evaluation

### Transpulmonary driving pressure

**Fig. 2:**
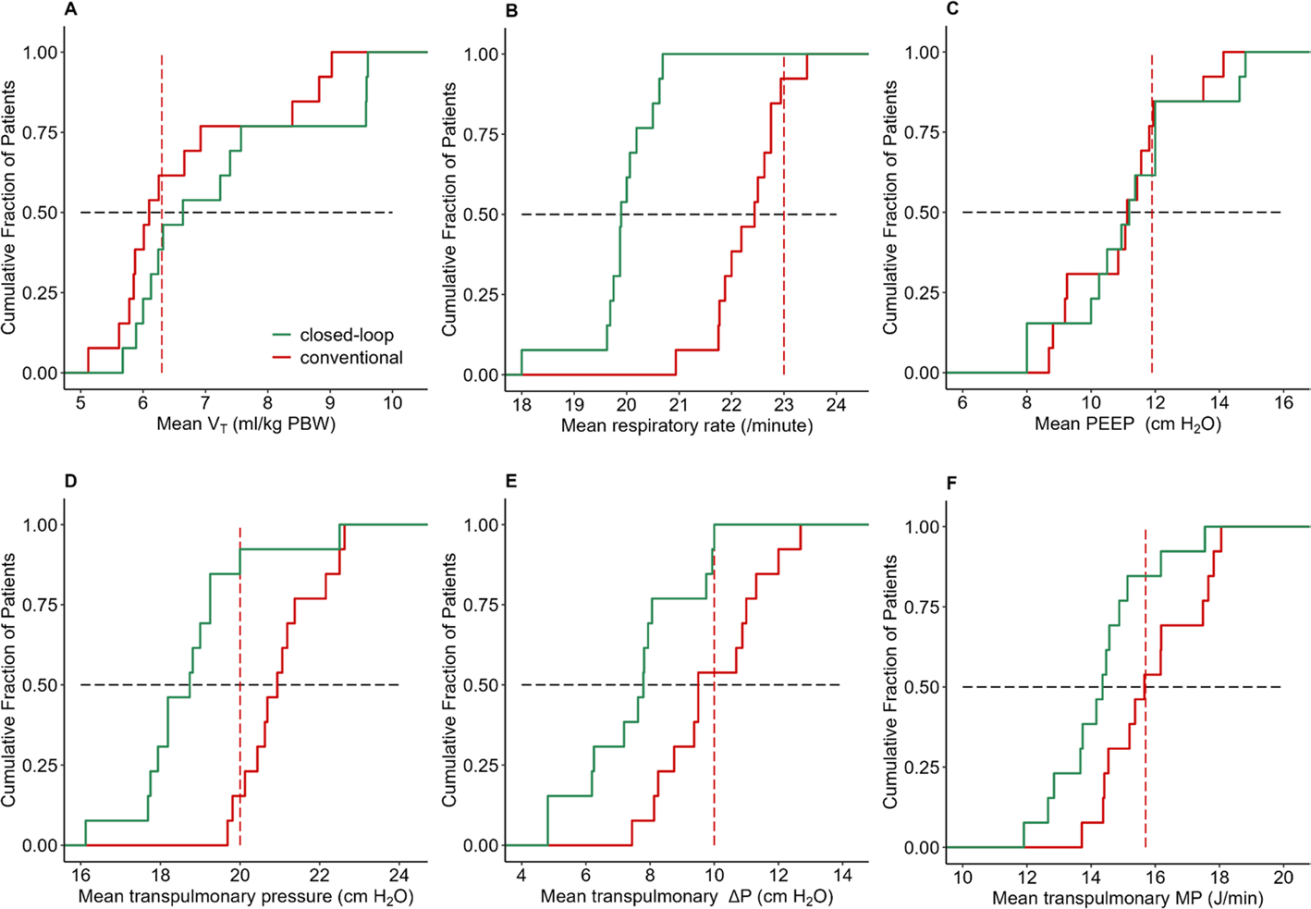
Cumulative frequency distribution of **A** *V*_T_, **B** RR, **C** PEEP, **D** inspiratory transpulmonary pressure, **E** transpulmonary Δ*P* and **F** transpulmonary MP. The plots show the mean variables with closed-loop ventilation and conventional ventilation. Vertical dotted lines represent the median value with conventional ventilation. Horizontal dotted lines show the respective proportion of patients reaching each cutoff

**Fig. 3:**
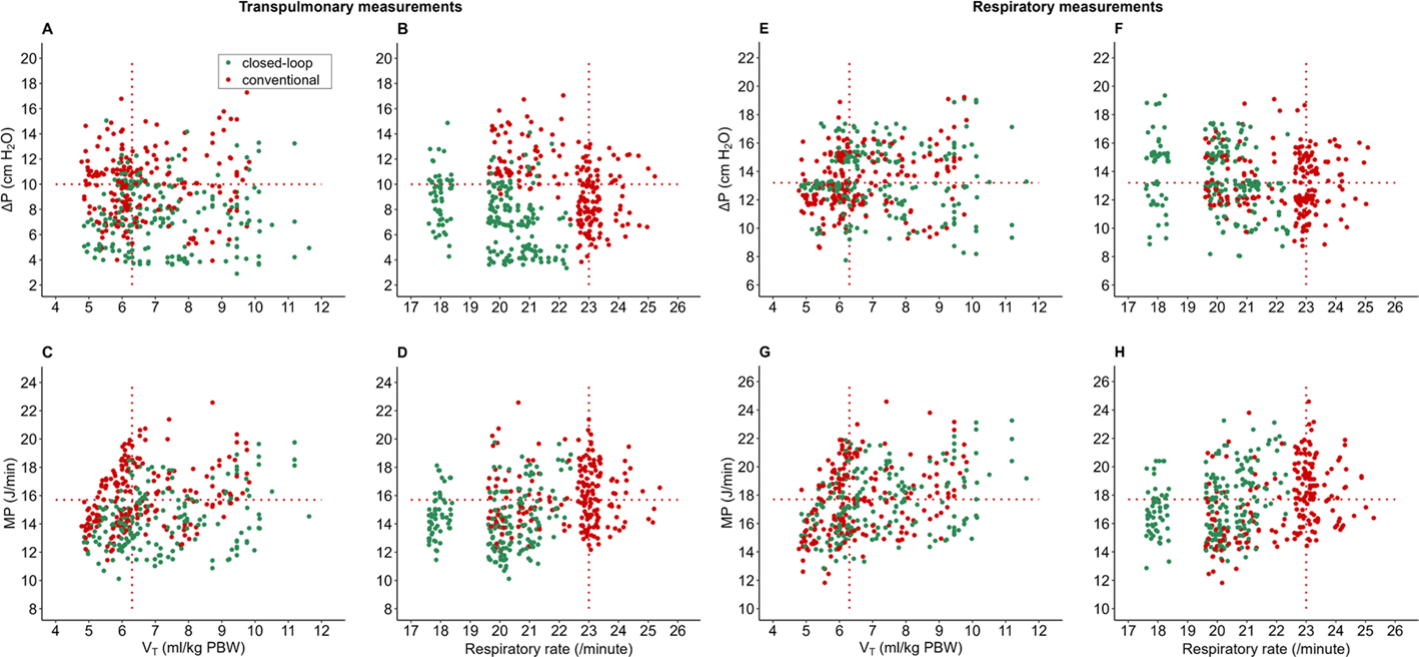
Scatterplots of transpulmonary measurements of the relationship of **A** *V*_T_ vs Δ*P*_TP_, **B** RR vs Δ*P*_TP_, **C** *V*_T_ vs MP_TP_ and **D** RR vs MP_TP_, and respiratory system measurements of **E** *V*_T_ vs Δ*P*_RS_, **F** RR vs Δ*P*_RS_, **G** *V*_T_ vs MP_RS_ and **H** RR vs MP_RS_, with closed-loop ventilation and conventional ventilation. Each time point of the individual patient was characterized by a single data point. Horizontal and vertical lines represent the median for the corresponding value with conventional ventilation. This figure visualizes the relation between the variables, and for instance shows that higher *V*_T_ does not necessarily translates in a higher Δ*P*_TP_ (**A**) or MP_TP_ (**C**) in individual patients. The horizontal and vertical lines based on median values in the current study create quadrants that could be used for interpretation of whether a certain combination is completely, or partially within safe zones of ventilation

Compared to conventional ventilation, with closed-loop ventilation the median Δ*P*_TP_ was lower (7.0 [5.0–10.0] versus 10.0 [8.0–11.0] cmH_2_O (mean difference − 2.5 [95% CI − 2.6 to − 2.1] cmH_2_O; *F* (1,11) = 33.204; *P* = 0.0001) (Table 2, Figs. 2 and 3). The ventilation mode had a significant effect which did not change with time (*P* = 0.15) nor with the interaction of ventilation mode * time (*P* = 0.78).

**Table 2:** Ventilation parameters

	INTELLiVENT-ASV	Conventional ventilation	*P* value
Primary endpoint			
∆*P*_TP_ (cmH_2_O)	7.0 (5.0–10.0)	10.0 (8.0–11.0)	0.0002
Secondary endpoint			
∆*P*_RS_ (cmH_2_O)	12.8 (12.0–15.0)	13.2 (12.0–15.0)	0.35
Pplat (cmH_2_O)	24.0 (24.0–25.0)	25.0 (24.0–26.0)	0.055
PEEP (cmH_2_O)	12.0 (10.0–12.0)	12.0 (9.0–12.0)	0.55
*P*_TP_ inspiratory	18.0 (17.0–20.0)	20.0 (19.0–23.0)	0.0002
*V*_T_ (mL)	498 (462–517)	453 (419–490)	0.003
*V*_T_ (mL/kg PBW)	6.9 (6.4–7.2)	6.3 (5.8–6.8)	0.002
RR (breaths/min)	20 (18–21)	23 (21–23)	< 0.001
Min. Vol (L/min)	9.8 (8.9–10.5)	10.1 (9.2–11.1)	0.03
*C*_RS_ (mL/cmH_2_O)	34.0 (31.0–41.0)	34.0 (30.0–39.0)	0.11
*C*_L_ (mL/cmH_2_O)	26.1 (23.5–29.7)	21.8 (19.6–24.3)	0.00027
*C*_CW_ (mL/cmH_2_O)	83.5 (67.8–96.1)	124.5 (99.6–186.5)	0.0003
*E*_L_ (cmH_2_O/L)	15.2 (11.3–19.1)	22.5 (16.7–26.5)	< 0.001
MP_TP_ (J/min)	14.1 (12.7–15.6)	15.7 (14.2–17.4)	0.0006
MP_RS_ (J/min)	17.1 (15.7–18.9)	17.7 (15.6–19.4)	0.51
FiO_2_ (%)	0.63 (0.50–0.70)	0.60 (0.50–0.67)	0.06
SpO_2_ (%)	94 (93–96)	96 (93–96)	0.07
etCO_2_ (kPa)	6.3 (5.3–6.7)	6.1 (5.8–6.7)	0.001
VR	1.9 (1.7–2.5)	2.3 (2.0–2.7)	0.095
Blood gas variables			
pH	7.31 (7.28–7.34)	7.32 (7.29–7.34)	0.79
pCO_2_ (kPa)	7.9 (6.7–8.4)	7.7 (7.1–8.0)	0.53
pO_2_ (kPa)	8.7 (7.9–9.6)	8.9 (8.1–9.9)	0.62
Bic (mmol/L)	20.0 (18.0–22.0)	21.0 (18.0–22.0)	0.43
Arterial sat. (%)	94 (93–96)	96 (93–96)	0.17

Data are median (IQR)

Δ*P*_TP_: transpulmonary driving pressure; Δ*P*_RS_: driving pressure of the respiratory system; MP: mechanical power; MP_TP_: transpulmonary mechanical power; Pplat: plateau pressure; *P*_TP_ inspiratory: inspiratory transpulmonary pressure; *V*_T_: tidal volume; PBW: predicted body weight; cmH_2_O: centimeters of water; RR: respiratory rate; *C*_RS_: compliance of the respiratory system; *C*_L_: compliance of the lung; *C*_CW_: compliance of the chest wall; *E*_L_: lung elastance; Vol: minute volume; FiO_2_: fraction of inspired oxygen; SpO_2_: pulse oximetry; etCO_2_: end-tidal carbon dioxide; VR: ventilatory ratio; kPa: kilopascal; Bic: bicarbonate

### Other ventilatory parameters

Compared to conventional ventilation, with closed-loop ventilation the median Δ*P*_RS_ was not different (Table 2, Figs. 2 and 3). With the closed-loop mode, median Δ*P*_RS_ was < 15 cmH_2_O in 9 out of 13 vs. 10 out of 13 patients with conventional ventilation (at 81% vs. 87% of all time points). While median *V*_T_ increased in 8 out of 13 patients, median RR decreased in 12 out of 13 patients (Additional file 1: Fig. S1) overall leading to a lower minute volume with closed-loop ventilation. *V*_T_ increased mainly with closed-loop ventilation when a patient had a higher *C*_RS_ (Additional file 1: Figs. S2 and S3)_._ A higher *V*_T_ did not lead to a higher Δ*P*_TP_ with closed-loop ventilation at most time points (Fig. 3) and *P*_TP_ was lower with closed-loop ventilation. There were no differences in median PEEP (Additional file 1: Fig. S4), FiO_2_ and *C*_RS_. Individual effects of the ventilation modes over time on Δ*P*_TP_, PEEP, *V*_T_ and RR are shown in Additional file 1: Figs. S5–S8.

Gas exchange was not affected, with no differences in PaO_2_ and PaCO_2_ between the two cross-over phases (Table 2). EtCO_2_ was higher with closed-loop ventilation, but SpO_2_ was not different. Pairwise comparisons of ventilatory parameters at the individual time points with the Bonferroni adjustment showed that they were not significant at all time points (Additional file 1: Table S1 and Figs. S9–S13).

### Post hoc analyses

While MP_RS_ was not different between closed-loop ventilation and conventional ventilation, median MP_TP_ was lower with closed-loop ventilation (Table 2, Figs. 2 and 3). The linear mixed model analysis did not change the findings of the primary analysis, meaning that Δ*P*_TP_ was lower with closed-loop ventilation and time as well as the interaction between ventilation and time of treatment was not significant. Thus, it is likely that the ventilation mode had a direct effect, which did not increase over time. The model is specified in Additional file 1: Table S2.

## Discussion

The findings of this physiological randomized cross-over clinical trial in a limited number of patients with moderate-to-severe ARDS can be summarized as follows: (i) compared to conventional ventilation, INTELLiVENT-ASV, a closed-loop ventilation mode that targets the lowest work and force of breathing, reduces Δ*P*_TP_ and *P*_TP_; (ii) increases *V*_T_; and (iii) reduces RR.

The study has several strengths. First, by using a cross-over design we were able to compare ventilation parameters between conventional with closed-loop ventilation wherein each patient served as his or her own control. This increased the statistical power of this relatively small study. Next, the study protocol was simple and strictly followed in all patients. All doctors and nurses were well-trained and experienced in applying lung-protective ventilation, skilled in using the closed-loop mode, and qualified in using the esophageal balloon catheter. Given that the cross-over periods lasted only 4 h, changes in ventilator parameters are most likely the result of the switch to the alternative ventilation mode, rather than changes in the patients’ lung conditions. Finally, we strictly followed a predefined statistical analysis plan, written before cleaning and closing of the database.

To our best knowledge, is this the first study that compares Δ*P*_TP_ between closed-loop and conventional ventilation. Using transpulmonary pressures, instead of respiratory system pressures, allowed us to reduce the ‘noise’ that comes from possible increases in chest wall elastance [27] and airway resistance. In other words, this approach allowed us to determine better the effects of this closed-loop mode designed to target the lowest work and force of breathing on lung stress [3, 18, 28]. A lower Δ*P*_TP_ suggests that ventilation is provided in a more lung-protective way, possibly reducing the risks for or extend of ventilator-induced lung injury (VILI) [29].

The findings of our study extend current knowledge regarding the tested closed-loop ventilation mode. While previous studies showed that this closed-loop ventilation mode results in a lower Δ*P*_RS_ and Δ*P*_TP_, thus far only Δ*P*_RS_ has been compared directly with conventional ventilation [11–13, 24]. The results of our study show that a switch to closed-loop ventilation results in fast changes in ventilator settings in a relatively short period. Of note, we studied patients in the early phase of ARDS. Usually, this is a period during which many interventions take place, meaning that there is little time for setting the ventilator properly. Closed-loop ventilation modes can support health care providers in providing lung-protective ventilation in this often-hectic phase.

The finding that *V*_T_ size increases while Δ*P* decreases is in line with the findings of previous investigations. Indeed, we and others recently showed similar changes when switching the ventilator from conventional ventilation to closed-loop ventilation [12, 13, 30, 31]. The algorithms underneath INTELLiVENT-ASV target the lowest work of breathing [9] and the lowest force of breathing [10]. The first leads to the ‘best’ combination for RR and *V*_T_, based on the expiratory time constant (RC_exp_): the RR is gradually reduced while the inspiratory pressure (Pinsp) is titrated up to achieve a minute volume that fits the patient best. It may seem surprising then to see that while *V*_T_ increases, Δ*P*_TP_ decreases. This apparent contradiction may be explained as follows. First, in our study, Δ*P*_TP_ decreases and *V*_T_ increases, because pulmonary compliance increased with closed-loop ventilation, meaning that lung mechanics improved. This physiological mechanism could be explained by the fact that PEEP is automatically adjusted with closed-loop ventilation. There was no difference in median PEEP, but the adjustments over time we visualize in individual patients could have led to recruitment or less overdistension, resulting in the best possible compliance and a lower Δ*P*_TP_. Second, one algorithm of INTELLiVENT-ASV allows for permissive hypercapnia, meaning that at higher pressures, the system chooses to target a higher end-tidal CO_2_. Consequently, the minute volume is reduced, and this goal is mainly reached through a reduction in RR, as shown in our study. This may effect Δ*P* as with a lower RR there is more time for gas exchange, and preventing wasted ventilation in patients with ARDS that have increased physiological dead space [32], as reflected by the lower ventilatory ratio in our study during closed-loop ventilation. Also, a lower RR can decrease stress and strain on lung tissue [33], because it is important to consider the level of stress and strain delivered with each breath (reflected by the Δ*P*), but also how often this is repeated (reflected by the RR). Some preclinical studies indicate that lowering the respiratory rate can reduce the risk of VILI [34–36].

Important to mention is that in 2 out of 13 patients *V*_T_ was > 8 mL/kg PBW with closed-loop ventilation. Of note, this was only the case in patients that were also receiving a *V*_T_ > 8 mL/kg PBW with conventional ventilation. Nevertheless, this is above the generally accepted safety limits for *V*_T_ [37]. Interestingly, in these patients the Δ*P* remained low at all times. This may be explained that ventilatory strategies with a lower *V*_T_ and higher RR may only be beneficial for patients with very low *C*_RS_ [38]. In contrast to patients with a not so low *C*_RS_, where a higher *V*_T_, with the benefit of a lower RR, can be acceptable as long as Δ*P* remains below < 15 cmH_2_O [39–41].

The post hoc analysis showed a decrease in MP_TP_. MP is the energy transferred from the ventilator to the respiratory system, and together with Δ*P* reflect the ‘intensity’ of ventilation. Not only the Δ*P*, but also the MP has been shown to have associations with outcomes [4, 5, 25, 42]. Taken together, the findings of our study suggest that the closed-loop mode of interest reduces the intensity of ventilation in most patients, with respect to all factors that have associations with worse outcomes—*V*_T_, plateau pressure, Δ*P* and RR. This is also reflected by the summary value, i.e., MP.

This study has limitations. Blinding of the doctors and nurses taking care of the patients was not possible because of the nature of the intervention. The analysis of collected data, though, was done by an investigator that was blinded for the randomization phase. Second, the study was stopped early, because of an increased use of ELS in patients with ARDS at the study site. However, the predefined sample-size was reached for the cross-over part of the study. As this was a single-center study, we may not generalize its findings. We stress, however, that the team of doctors and nurses were experienced in applying lung-protective ventilation, which may not be the case everywhere. From the individual data we learned that not all patients respond in the same way to a switch between the two modes—individual patient responses need further attention in future studies. Last but not least, it is attractive to think and perhaps even plausible that a decrease in Δ*P* translates into clinical benefits, but this remains to be proven in future studies.

## Conclusion

In patients in the early phase of moderate-to-severe ARDS, a closed-loop mode that targets the lowest work and force of breathing decreases the transpulmonary Δ*P* in this small physiological study. Use of this mode also lowered RR, and MP. Future studies remain needed to determine if these changes provide clinical benefits.

## Supplementary Information

**Additional file 1: Table S1.** List of significant pairwise comparisons per time point, significance was determined at a *P* value < 0.003. **Table S2.** Specification of the generalized linear mixed model analysis. **Figure S1.** Line plots showing the mean changes and individual changes over time of transpulmonary Δ*P*, *V*_T_ and RR during the two study blocks of the two ventilation modes in the study. **Figure S2.** *C*_RS_ vs. *V*_T_ between closed-loop ventilation and conventional ventilation, and transpulmonary Δ*P* vs. *V*_T_ between closed-loop ventilation and conventional ventilation. A negative value means that the parameter decreased with closed-loop ventilation, and a higher value means that the parameter increased with closed-loop ventilation. All dots represent the mean value of an individual patient. **Figure S3.** Scatterplots of *C*_RS_ vs. *V*_T_ and transpulmonary Δ*P* vs. *V*_T_ with closed-loop ventilation and conventional ventilation. Each dot was characterized by a single data point. **Figure S4.** Showing individual patient data of the effect of the change of the ventilation mode on PEEP setting. **Figure S5.** Showing transpulmonary Δ*P* per patient during every time point, with closed-loop ventilation and conventional ventilation. The head with number represents the corresponding patient, the *x*-axis represent the 16 time points per block. **Figure S6.** Showing PEEP per patient during every time point, with closed-loop ventilation and conventional ventilation. The head with number represents the corresponding patient, the *x*-axis represent the 16 time points per block. **Figure S7.** Showing the tidal volumeper patient during every time point, with closed-loop ventilation and conventional ventilation. The head with number represents the corresponding patient, the *x*-axis represent the 16 time points per block. **Figure S8.** Showing the respiratory rateper patient during every time point, with closed-loop ventilation and conventional ventilation. The head with number represents the corresponding patient, the *x*-axis represent the 16 time points per block. **Figure S9.** Violin plot of pairwise comparisons at individual time points of Δ*P*. **Figure S10.** Violin plot of pairwise comparisons at individual time points of transpulmonary Δ*P*. **Figure S11.** Violin plot of pairwise comparisons at individual time points of RR. **Figure S12.** Violin plot of pairwise comparisons at individual time points of MP. **Figure S13.** Violin plot of pairwise comparisons at individual time points of transpulmonary MP.

## Data Availability

The dataset is available from the corresponding author on reasonable request.
