# Supplementary material for: A closed-loop ventilation mode that targets the lowest work and force of breathing reduces the transpulmonary driving pressure in patients with moderate-to-severe ARDS
Source: Intensive Care Med Exp. 2023 Jul 14;11:42. doi: 10.1186/s40635-023-00527-1 (PMC10344850; doi:10.1186/s40635-023-00527-1)
Supplement: Supplementary file 1 — Additional file 1: Table S1. List of significant pairwise comparisons per time point, significance was determined at a P value < 0.003. Table S2. Specification of the generalized linear mixed model analysis. Figure S1. Line plots showing the mean changes and individual changes over time of transpulmonary ΔP, VT and RR during the two study blocks of the two ventilation modes in the study. Figure S2. CRS vs. VT between closed-loop ventilation and conventional ventilation, and transpulmonary ΔP vs. VT between closed-loop ventilation and conventional ventilation. A negative value means that the parameter decreased with closed-loop ventilation, and a higher value means that the parameter increased with closed-loop ventilation. All dots represent the mean value of an individual patient. Figure S3. Scatterplots of CRS vs. VT and transpulmonary ΔP vs. VT with closed-loop ventilation and conventional ventilation. Each dot was characterized by a single data point. Figure S4. Showing individual patient data of the effect of the change of the ventilation mode on PEEP setting. Figure S5. Showing transpulmonary ΔP per patient during every time point, with closed-loop ventilation and conventional ventilation. The head with number represents the corresponding patient, the x-axis represent the 16 time points per block. Figure S6. Showing PEEP per patient during every time point, with closed-loop ventilation and conventional ventilation. The head with number represents the corresponding patient, the x-axis represent the 16 time points per block. Figure S7. Showing the tidal volumeper patient during every time point, with closed-loop ventilation and conventional ventilation. The head with number represents the corresponding patient, the x-axis represent the 16 time points per block. Figure S8. Showing the respiratory rateper patient during every time point, with closed-loop ventilation and conventional ventilation. The head with number represents the corresponding patient, the x-axis [file 40635_2023_527_MOESM1_ESM.docx]

**Additional file 1**

**A Closed-loop Ventilation Mode that Targets the Lowest Work and Force of Breathing Reduces the Transpulmonary Driving Pressure in Subjects with moderate-to-severe ARDS—a singlecenter randomized crossover clinical trial**

Laura A. Buiteman-Kruizinga, David M.P. van Meenen, Lieuwe D.J. Bos,

Pim L.J. van der Heiden, Frederique Paulus_,_ Marcus J. Schultz

**Table of contents**

**Additional Tables**

Table S1 3

Table S2 4

**Additional Figures**

Figure S1 5

Figure S2 6

Figure S3 7

Figure S4 8

Figure S5 9

Figure S6 10

Figure S7 11

Figure S8 12

Figure S9 13

Figure S10 14

Figure S11 15

Figure S12 16

Figure S13 17

**Additional Tables**

| **Table S1.** List of significant pairwise comparisons per time point, significance was determined at a *P* value < 0.003 | |
| --- | --- |
|  | n = 13 |
| ΔP_RS_ (cm H_2_O) | None |
| ΔP_TP_ (cm H_2_O) | 2,3,7,9,10 |
| P_TPinsp_ | 1,2,3,5,7,8,9,11 |
| V_T_ (ml/kg PBW) | 2,5,6,9,11,12,13,15 |
| RR (/min) | All |
| MP_RS_ (J/min) | None |
| MP_TP_ (J/min) | 1,3,4,5,8,10 |
| ΔP_RS_: driving pressure of the respiratory system; ΔP_TP_: transpulmonary driving pressure; P_TPinsp_: inspiratory transpulmonary pressure; V_T_: tidal volume; PBW: predicted body weight; cm H_2_O: centimeters of water; RR: respiratory rate; MP_RS_: mechanical power of the respiratory system; MP_TP_: transpulmonary mechanical power | |

| **Table S2.** Specification of the generalized linear mixed model analysis | | | |
| --- | --- | --- | --- |
|  | Fixed effects | 95% CI | *P* value |
| Primary endpoint |  |  |  |
| ΔP_TP_ (cm H_2_O) | Mode 1.41  Time point –0.07  Mode * time point –0.03 | 0.99 to 1.84  –0.12 to 0.03  –0.07 to 0.02 | < 0.001  0.15  0.249 |
| Secondary endpoint | | | |
| ΔP_RS_ (cm H_2_O) | Mode –0.15  Time point –0.05  Mode * time point –0.007 | –0.46 to 0.17  –0.08 to 0.015  –0.04 to 0.03 | 0.36  0.06  0.68 |
| V_T_ (mL/kg PBW) | Mode –0.25  Time point –0.013  Mode * time point –0.004 | –0.38 to –0.12  –0.03 to 0.0006  –0.02 to 0.009 | 0.0002  0.06  0.557 |
| RR (breaths / min) | Mode 1.17  Time point 0.003  Mode * time point 0.002 | 0.93 to 1.42  0.004 to 0.06  –0.02 to 0.03 | < 0.001  0.0225  0.82 |
| MP_TP_ (J/min) | Mode 1.42  Time point –0.02  Mode * time point –0.04 | 0.82 to 1.47  –0.05 to 0.02  –0.08 to –0.008 | < 0.001  0.3003  0.0162 |
| MP_RS_ (J/min) | Mode 0.44  Time point –0.008  Mode * time point –0.04 | 0.06 to 0.83  –0.05 to 0.03  –0.08 to 0.005 | 0.024  0.68  0.09 |
| Abbreviations: ΔP_RS_: respiratory system driving pressure; ΔP_TP_: transpulmonary driving pressure; MP_RS_: mechanical power of the respiratory system; MP_TP_: transpulmonary mechanical power; V_T_: tidal volume; PBW: predicted body weight; cm H_2_O: centimeters of water; RR: respiratory rate; CI: confidence interval | | | |

**Additional Figures**

**
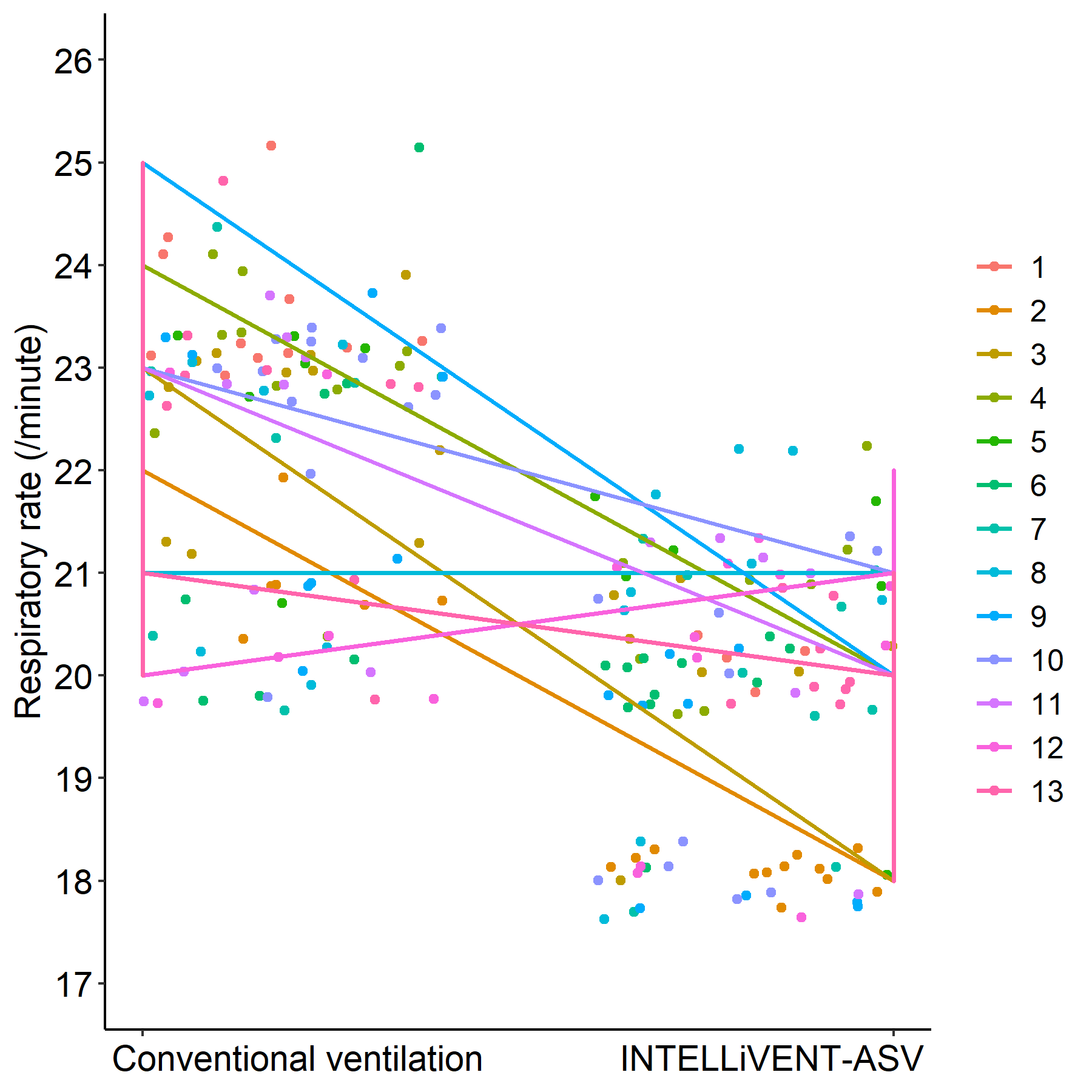

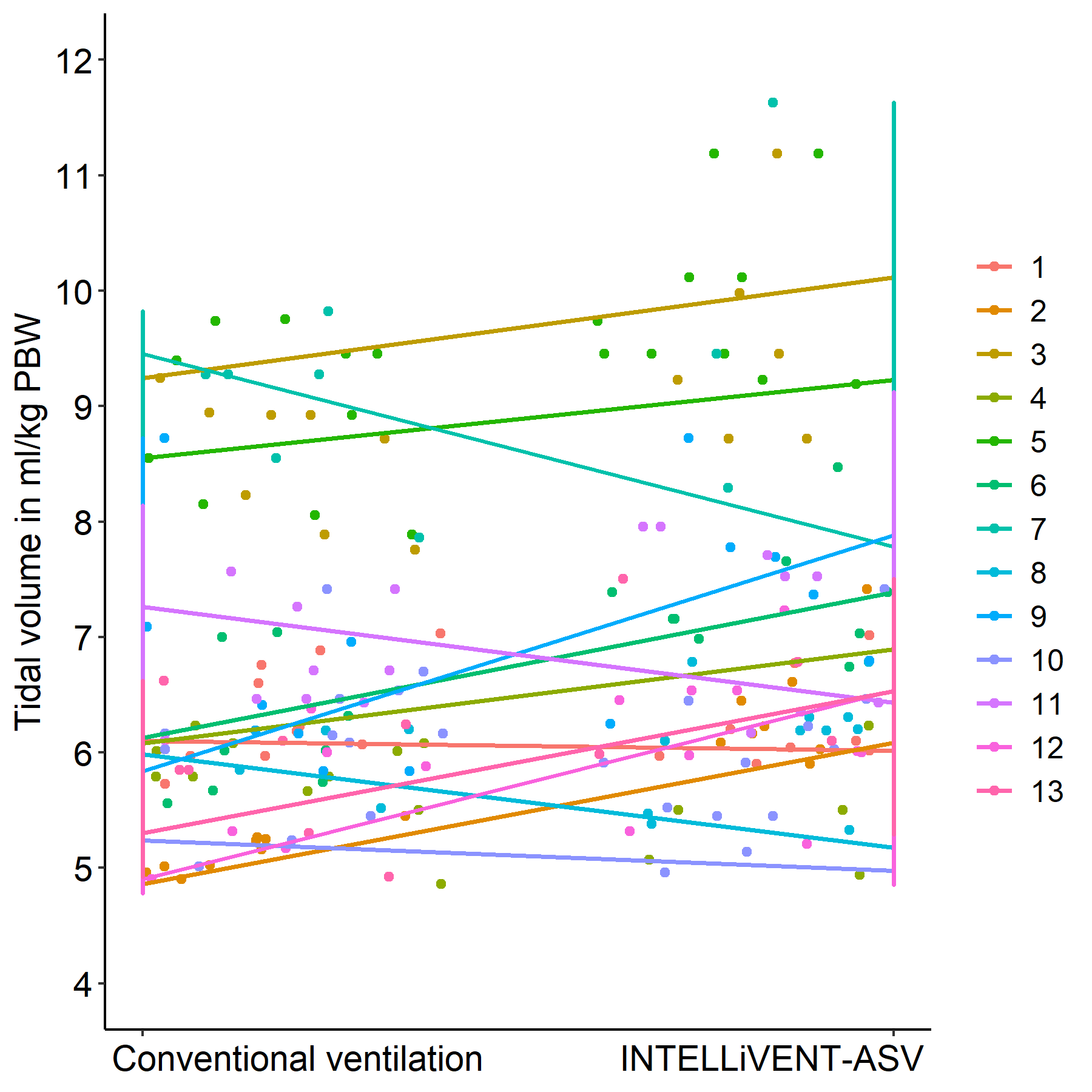

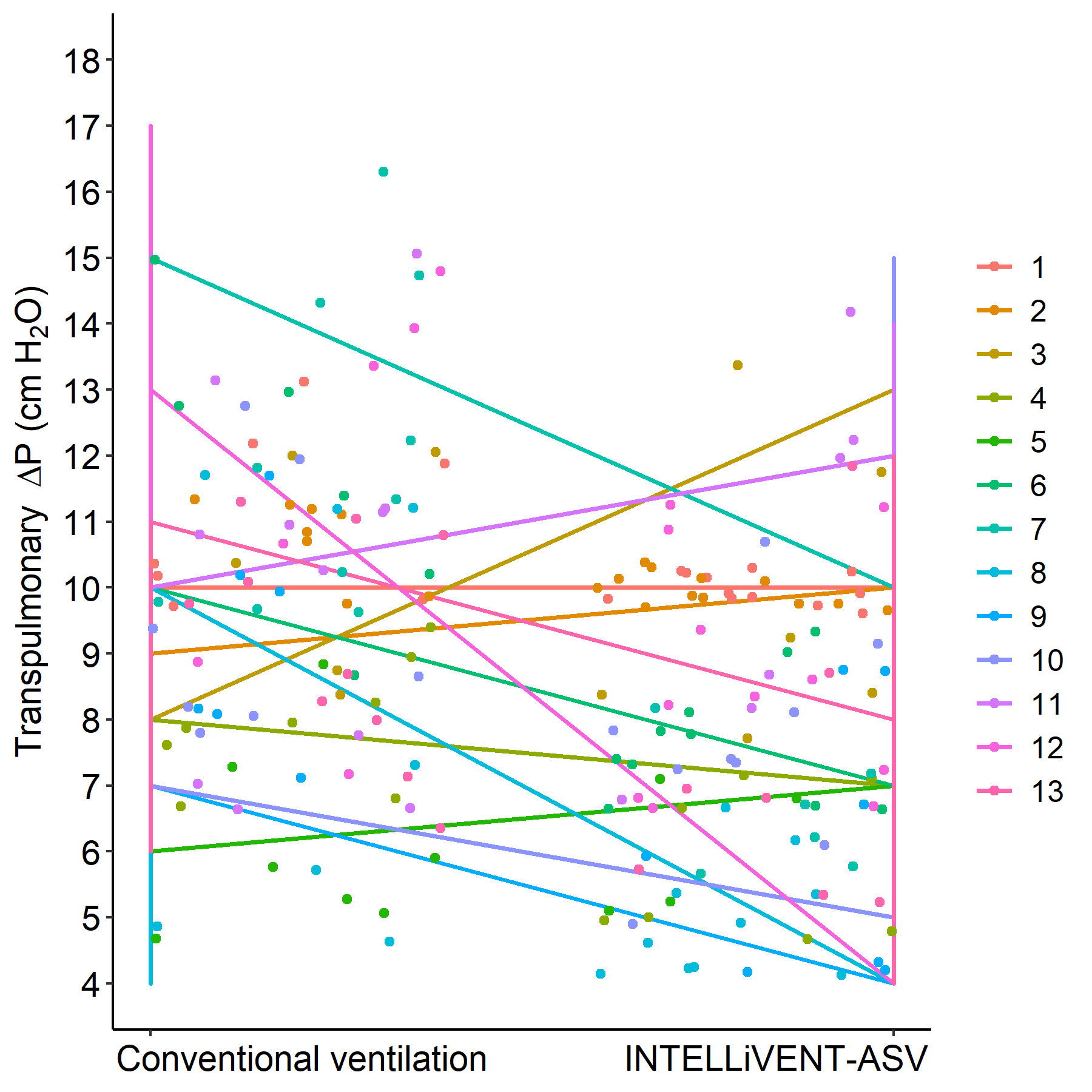
Figure S1**

**Figure S1.** Line plots showing the mean changes and individual changes over time of transpulmonary ΔP, V_T_ and RR during the two study blocks of the two ventilation modes in the study.

**Figure S2**

**
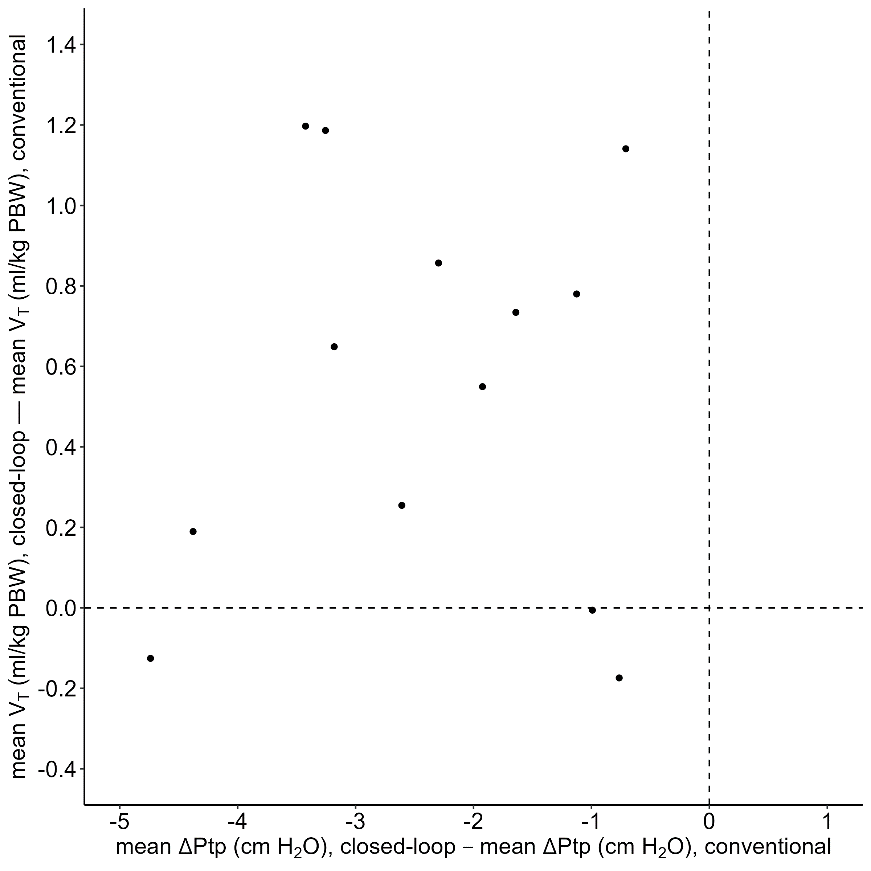

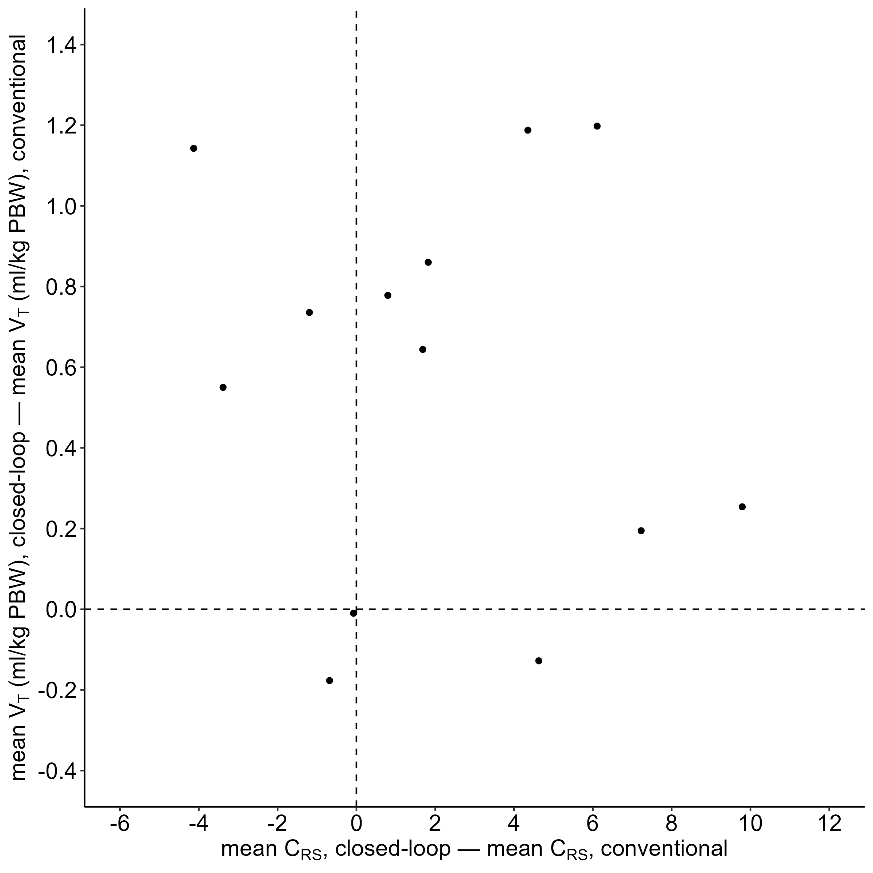
**

**Figure S2.** C_RS_ *vs* V_T_ between closed-loop ventilation and conventional ventilation, and transpulmonary ΔP *vs* V_T_ between closed-loop ventilation and conventional ventilation. A negative value means that the parameter decreased with closed-loop ventilation, and a higher value means that the parameter increased with closed-loop ventilation. All dots represent the mean value of an individual patient.

**Figure S3**

**
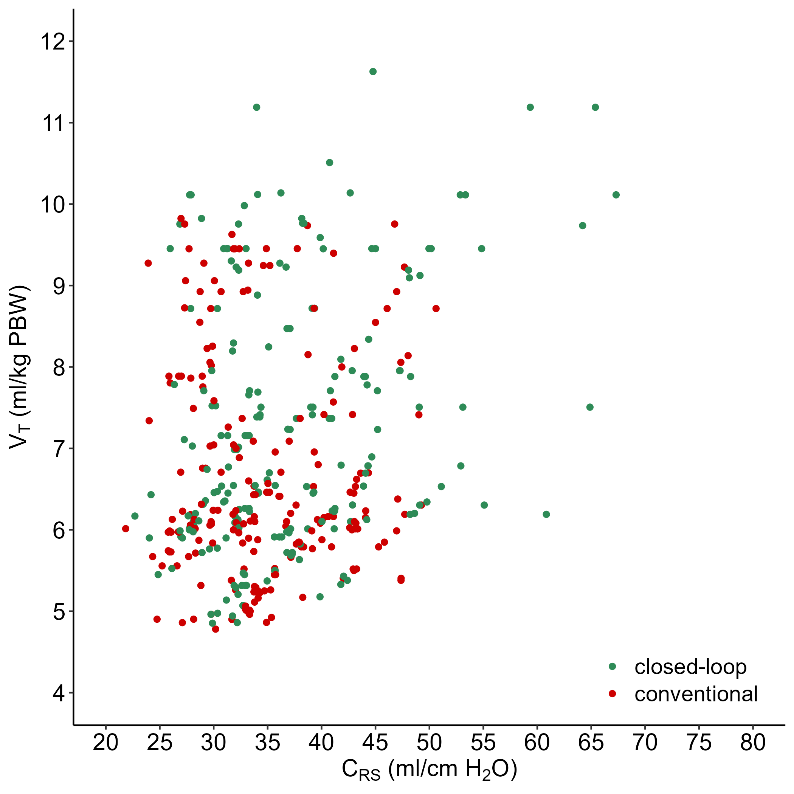

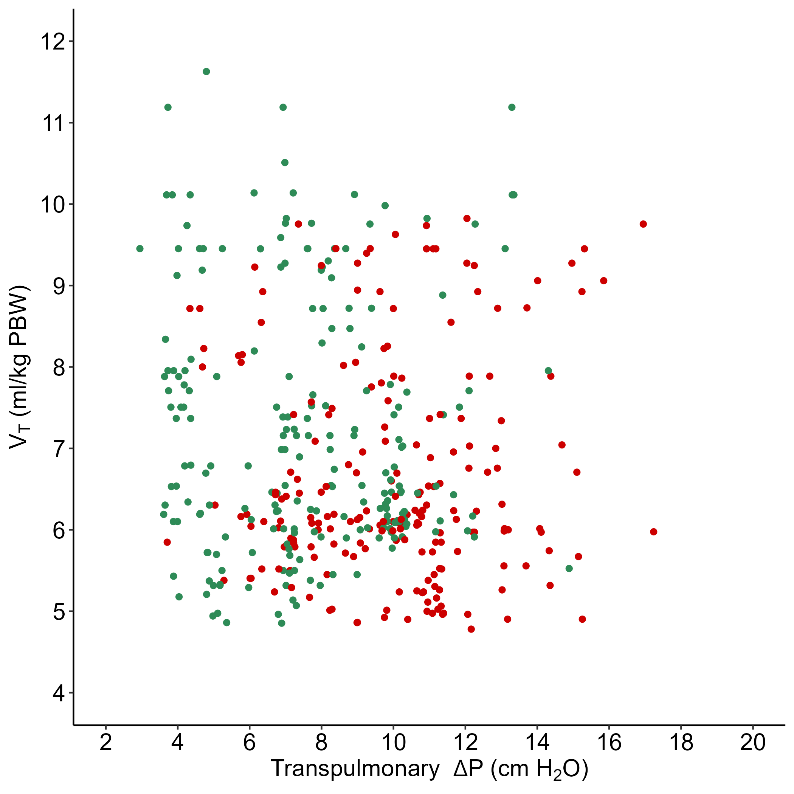
**

**Figure S3.** Scatterplots of C_RS_ *vs* V_T_ and transpulmonary ΔP *vs* V_T_ with closed–loop ventilation and conventional ventilation. Each dot was characterized by a single data point.

**Figure S4**

**
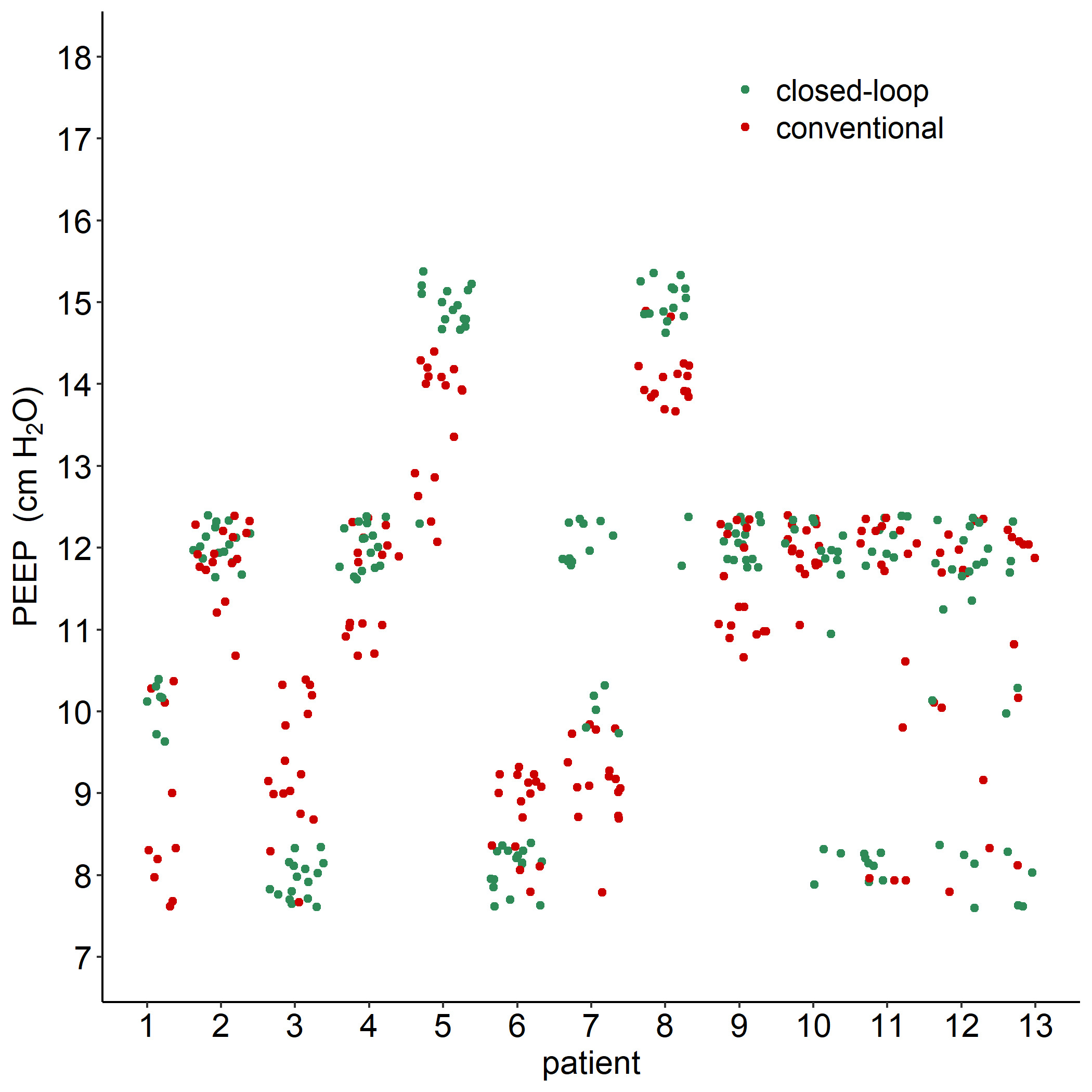
**

**Figure S4.** Showing individual patient data of the effect of the change of the ventilation mode on PEEP setting.

**Figure S5**

**
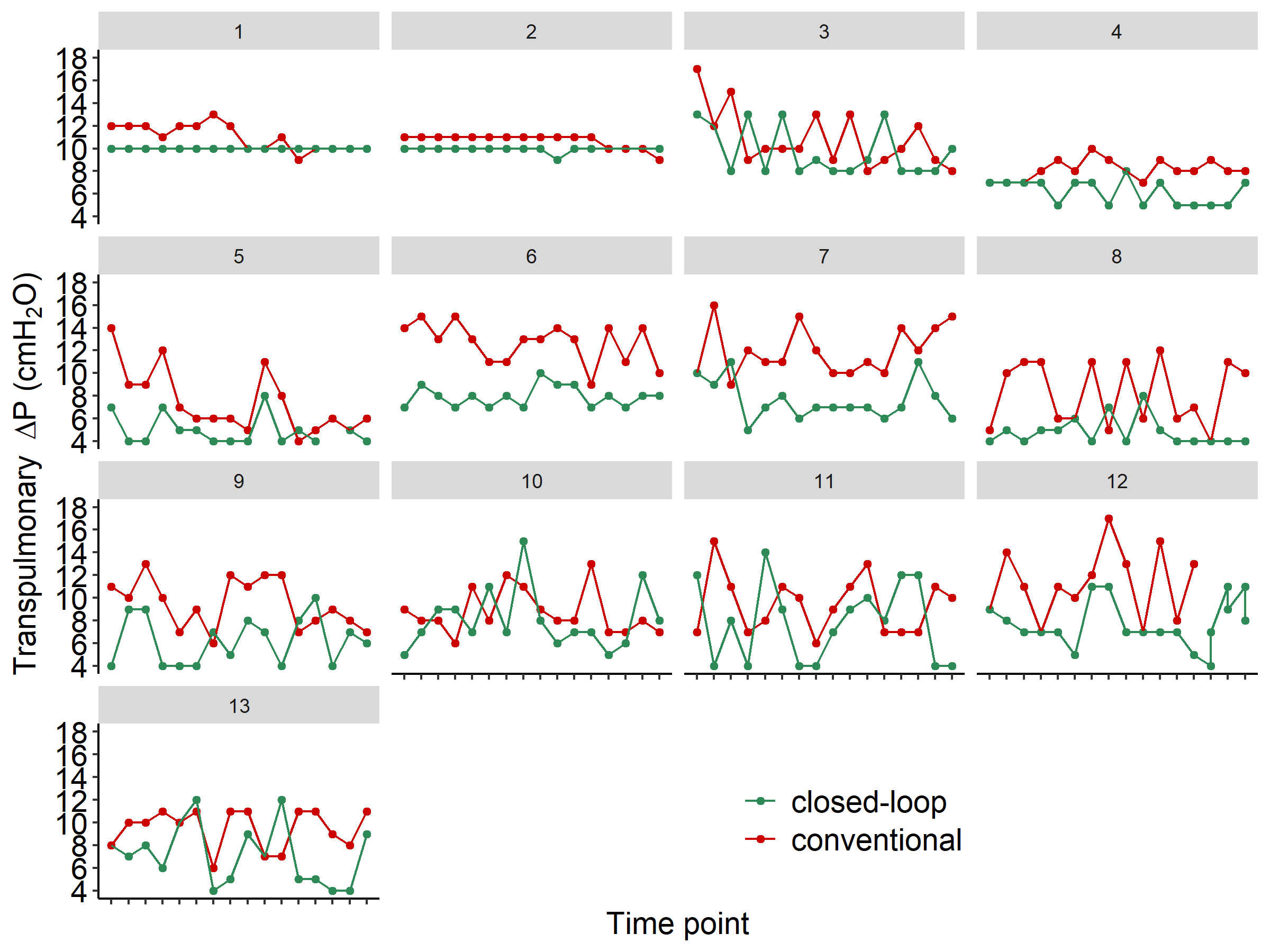
**

**Figure S5.** Showing transpulmonary ΔP per patient during every time point, with closed−loop ventilation and conventional ventilation. The head with number represents the corresponding patient, the x-axis represent the 16 time points per block.

**Figure S6**


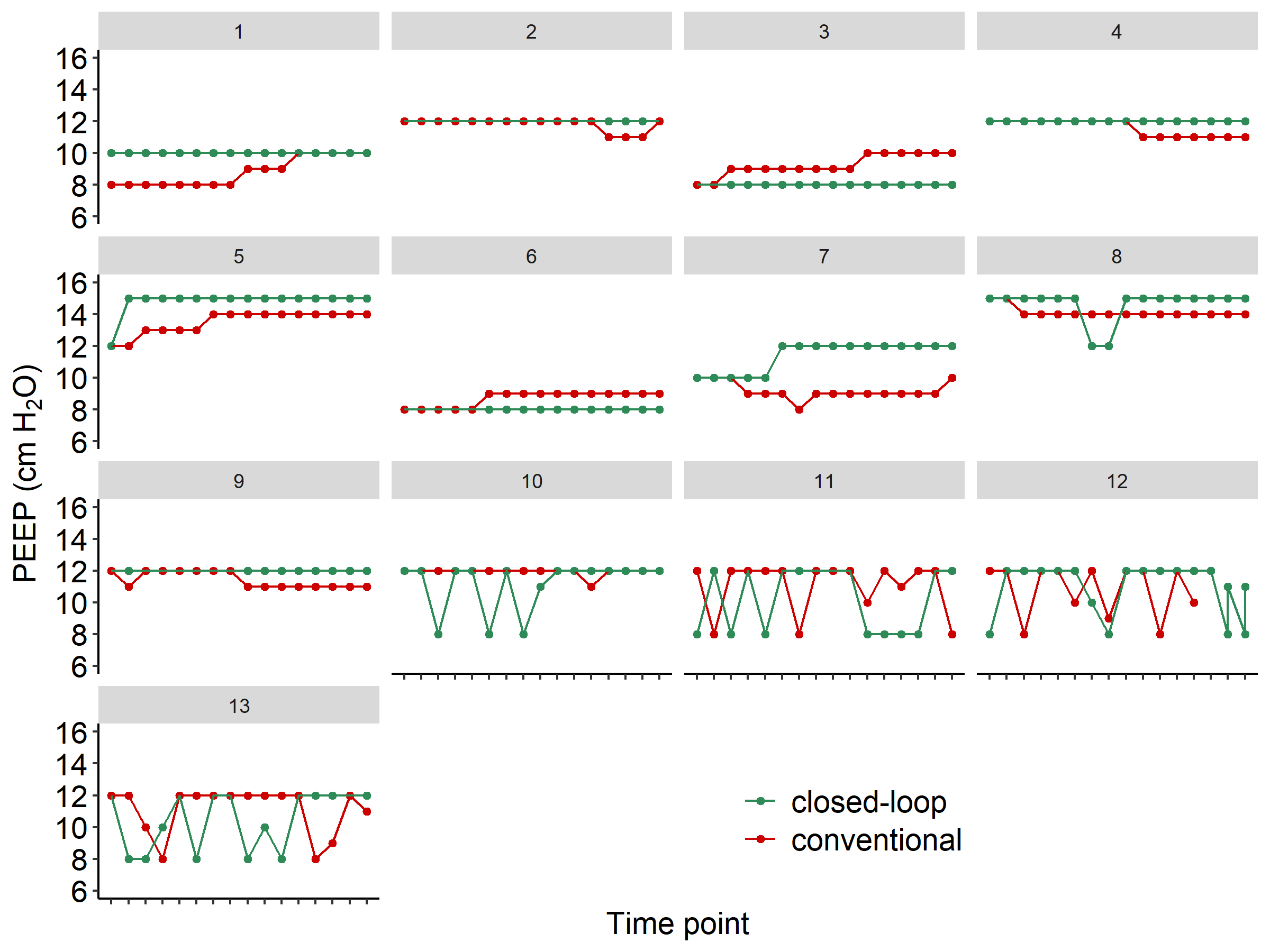


**Figure S6.** Showing PEEP per patient during every time point, with closed−loop ventilation and conventional ventilation. The head with number represents the corresponding patient, the x-axis represent the 16 time points per block.

**Figure S7**

**
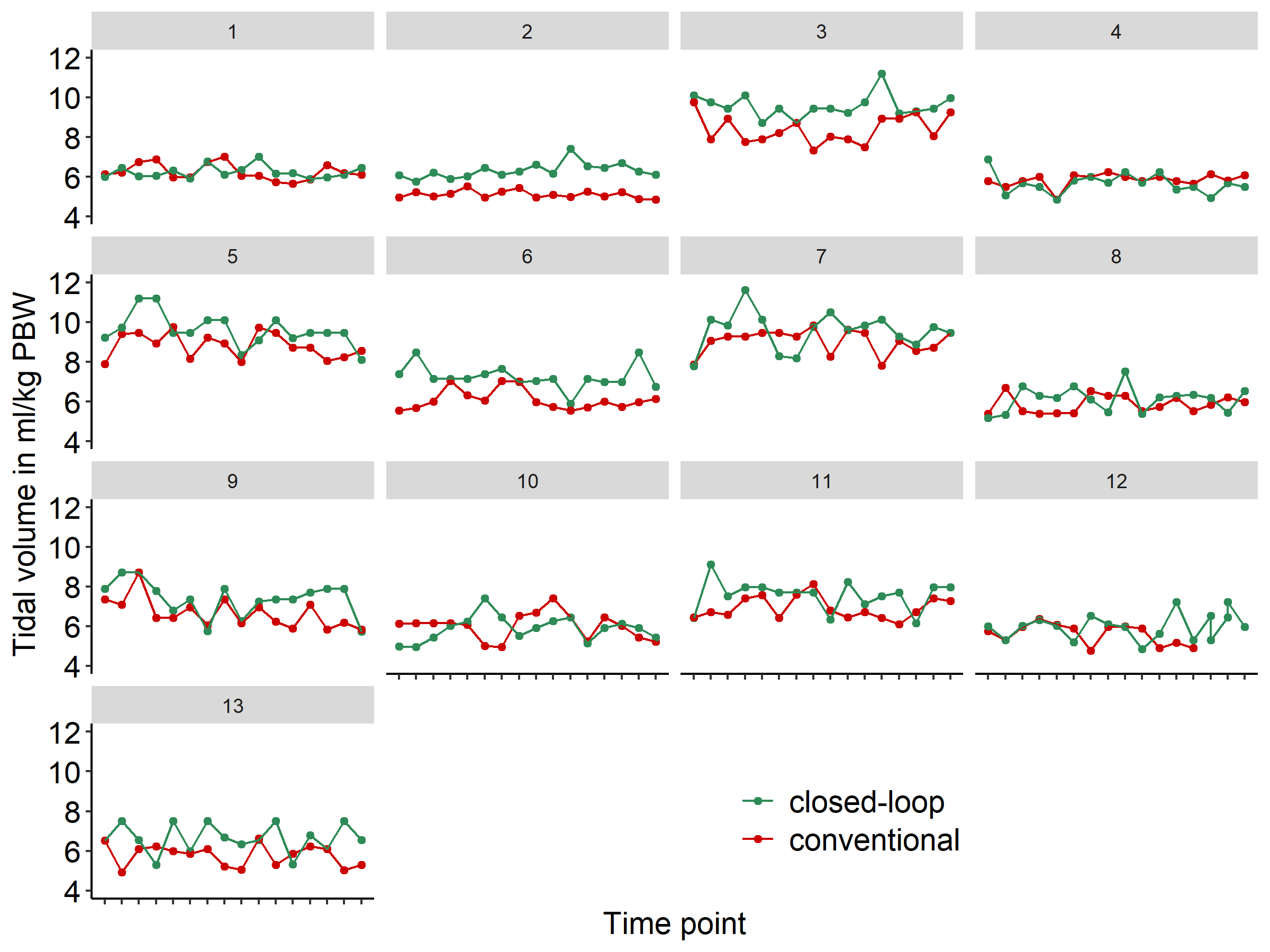
**

**Figure S7.** Showing the tidal volume (ml/kg PBW) per patient during every time point, with closed−loop ventilation and conventional ventilation. The head with number represents the corresponding patient, the x-axis represent the 16 time points per block.

**Figure S8**

**
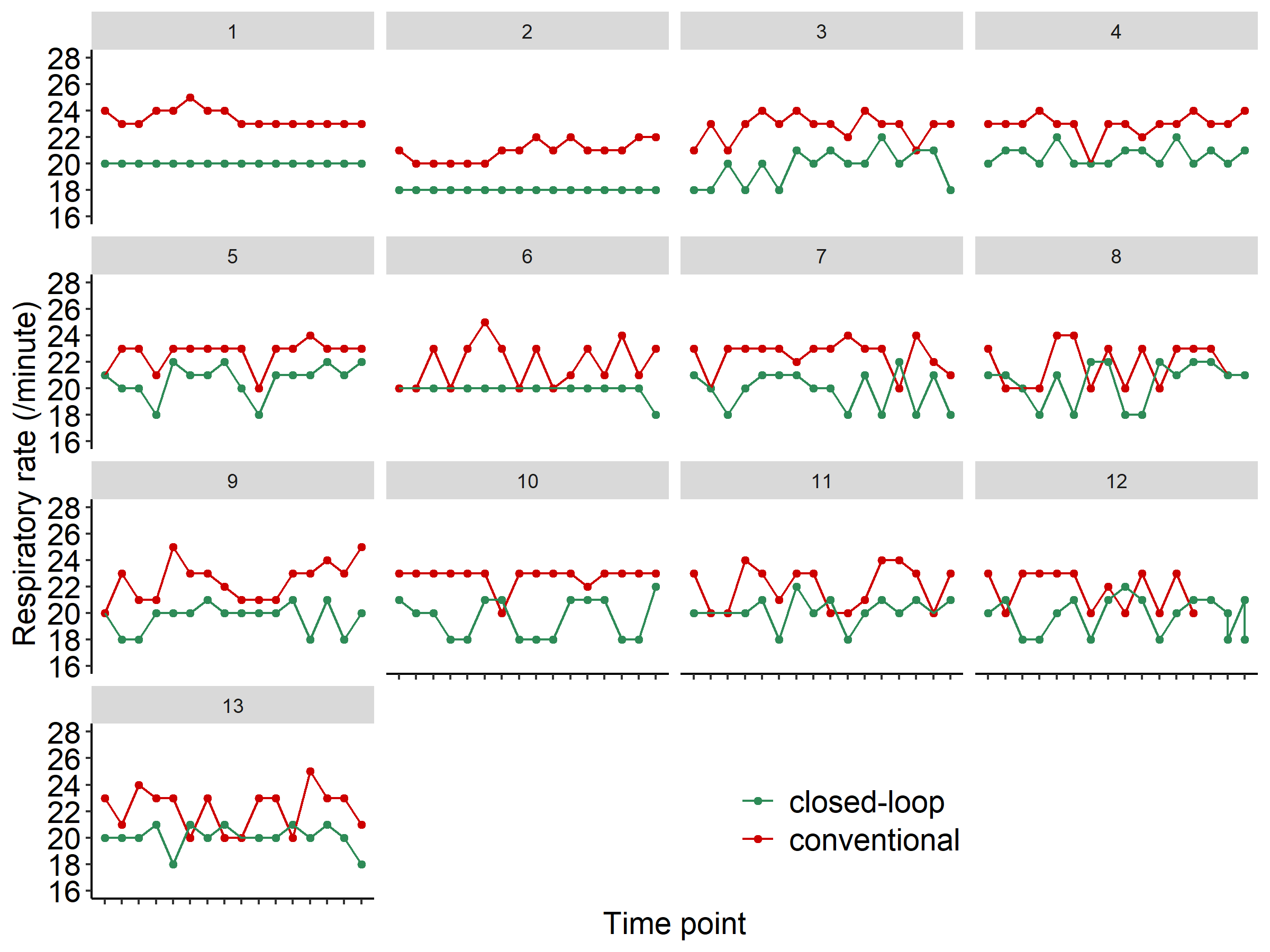
**

**Figure S8.** Showing the respiratory rate (per minute) per patient during every time point, with closed−loop ventilation and conventional ventilation. The head with number represents the corresponding patient, the x-axis represent the 16 time points per block.

**Figure S9**

**
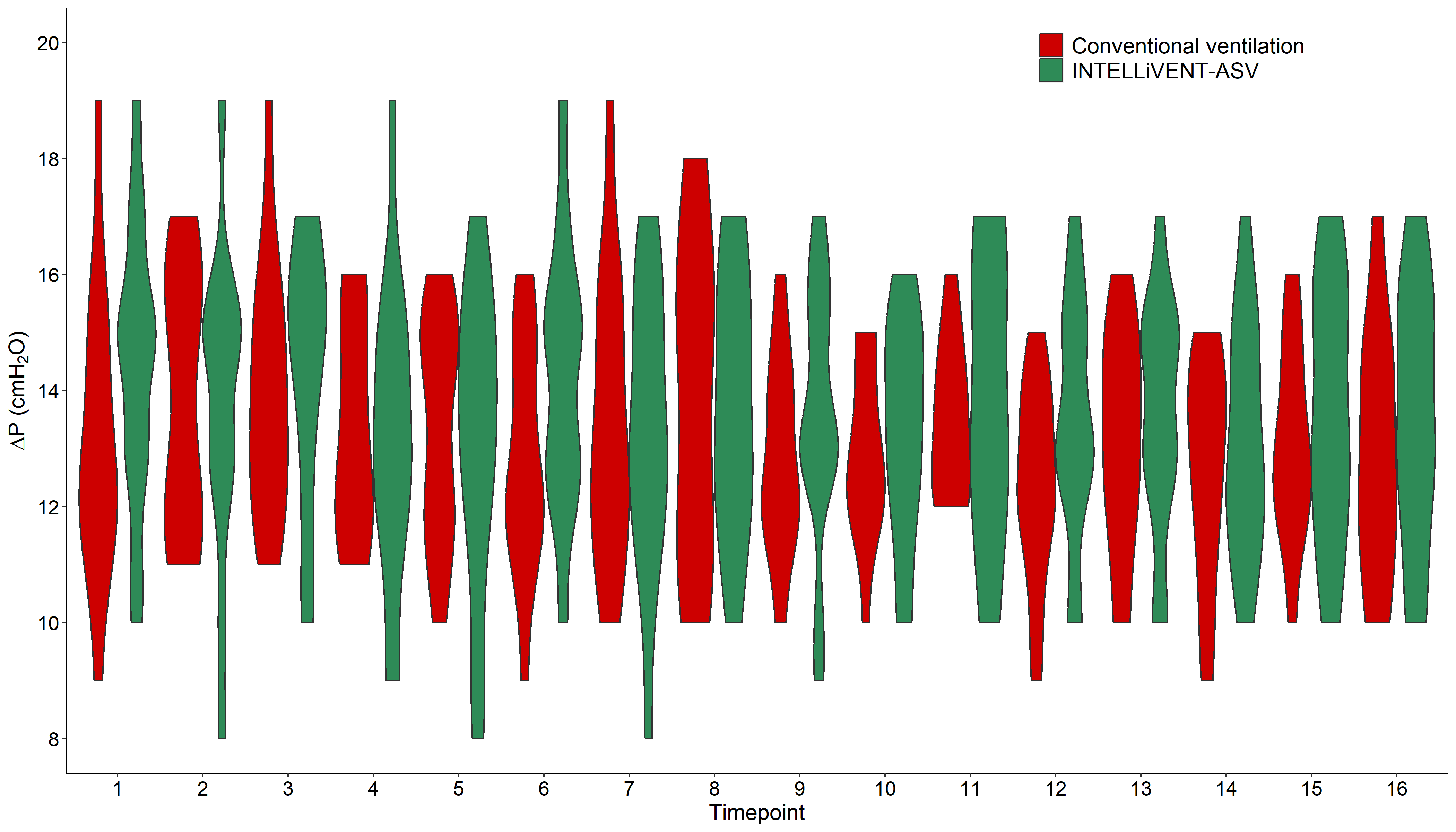
**

**Figure S9.** Violin plot of pairwise comparisons at individual time points of ΔP.

**Figure S10**

**
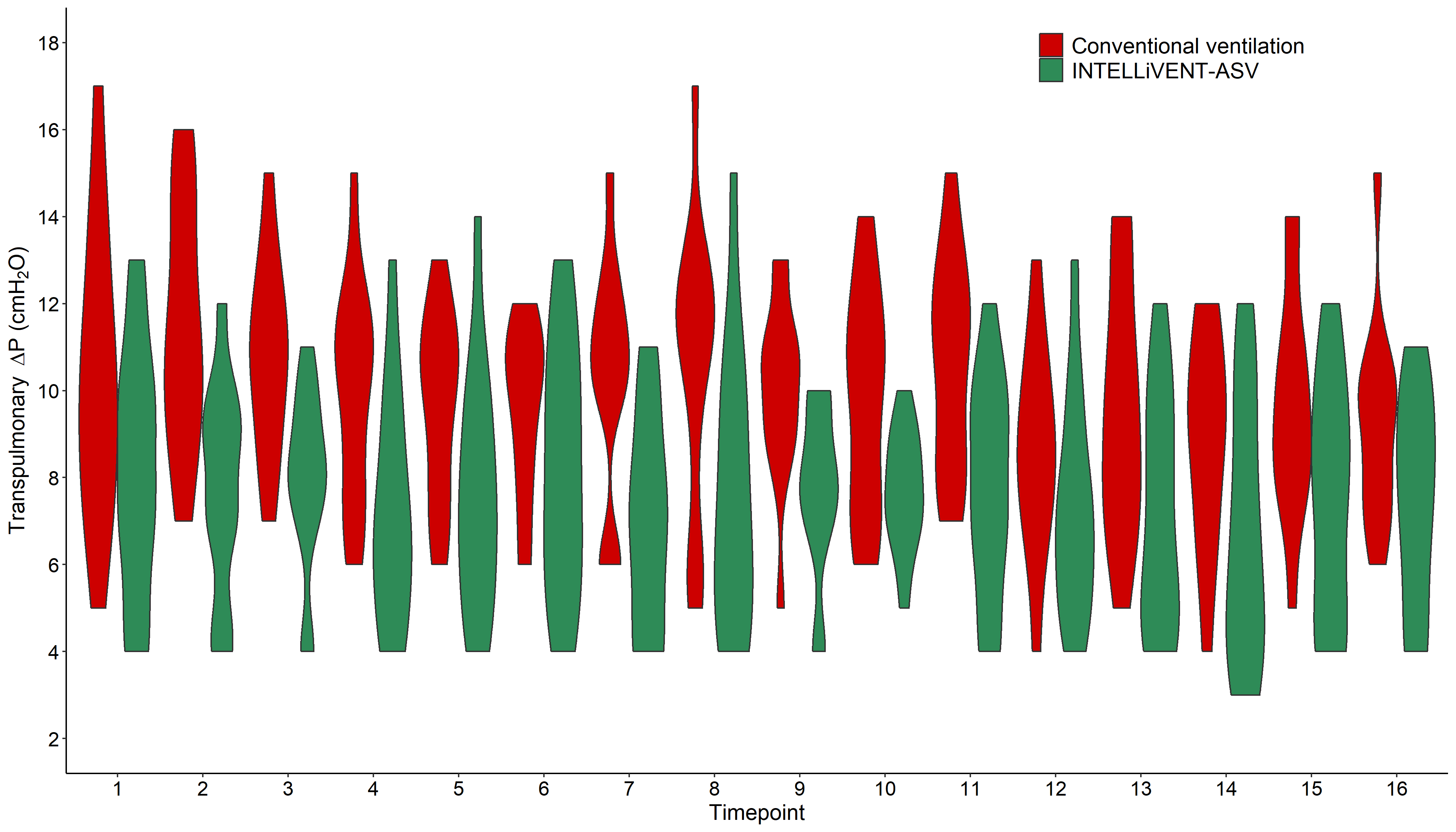
**

**Figure S10.** Violin plot of pairwise comparisons at individual time points of transpulmonary ΔP.

**Figure S11**

**
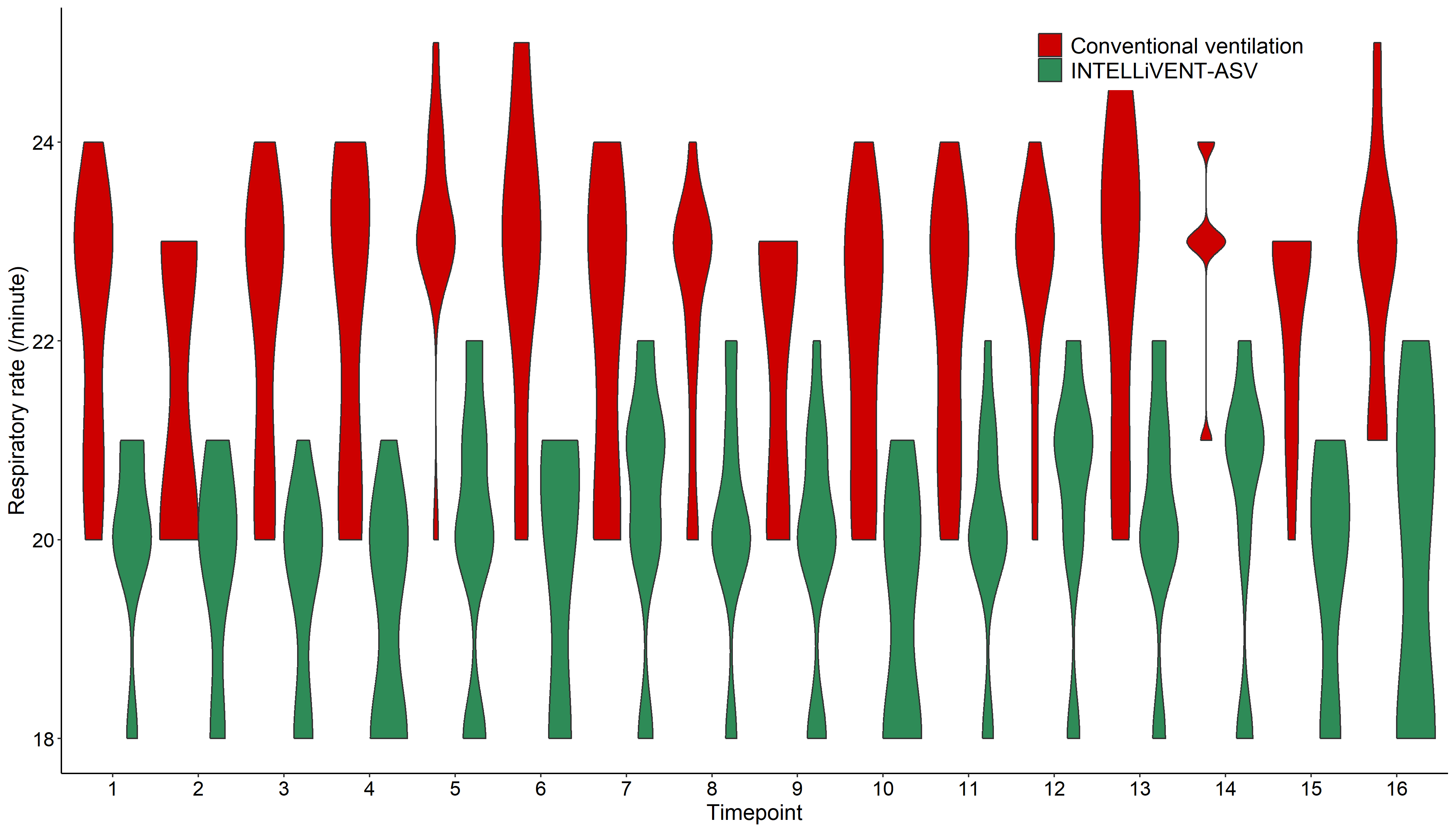
**

**Figure S11.** Violin plot of pairwise comparisons at individual time points of RR.

**
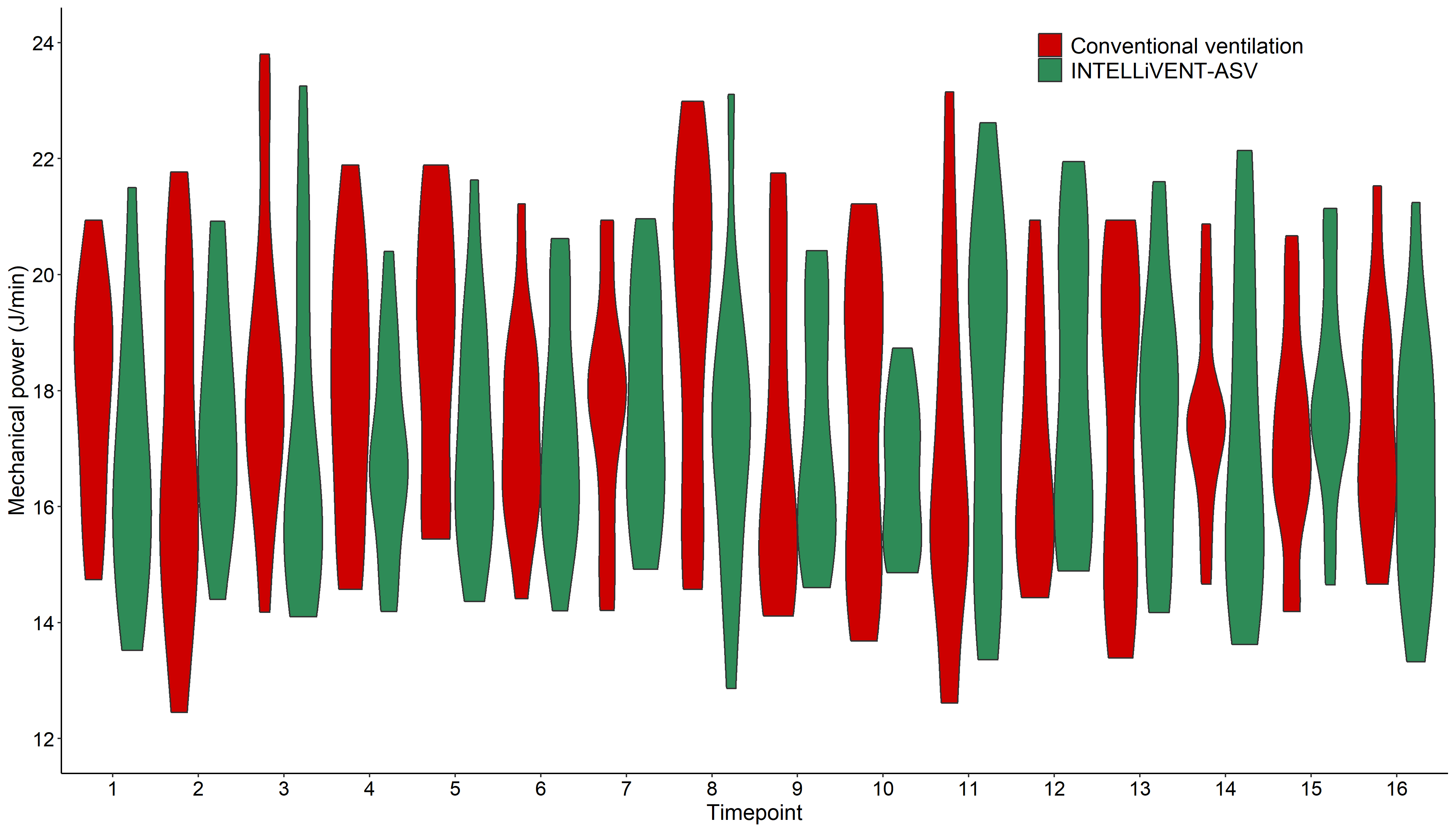
Figure S12**

**Figure S12.** Violin plot of pairwise comparisons at individual time points of MP.

**Figure S13**

**
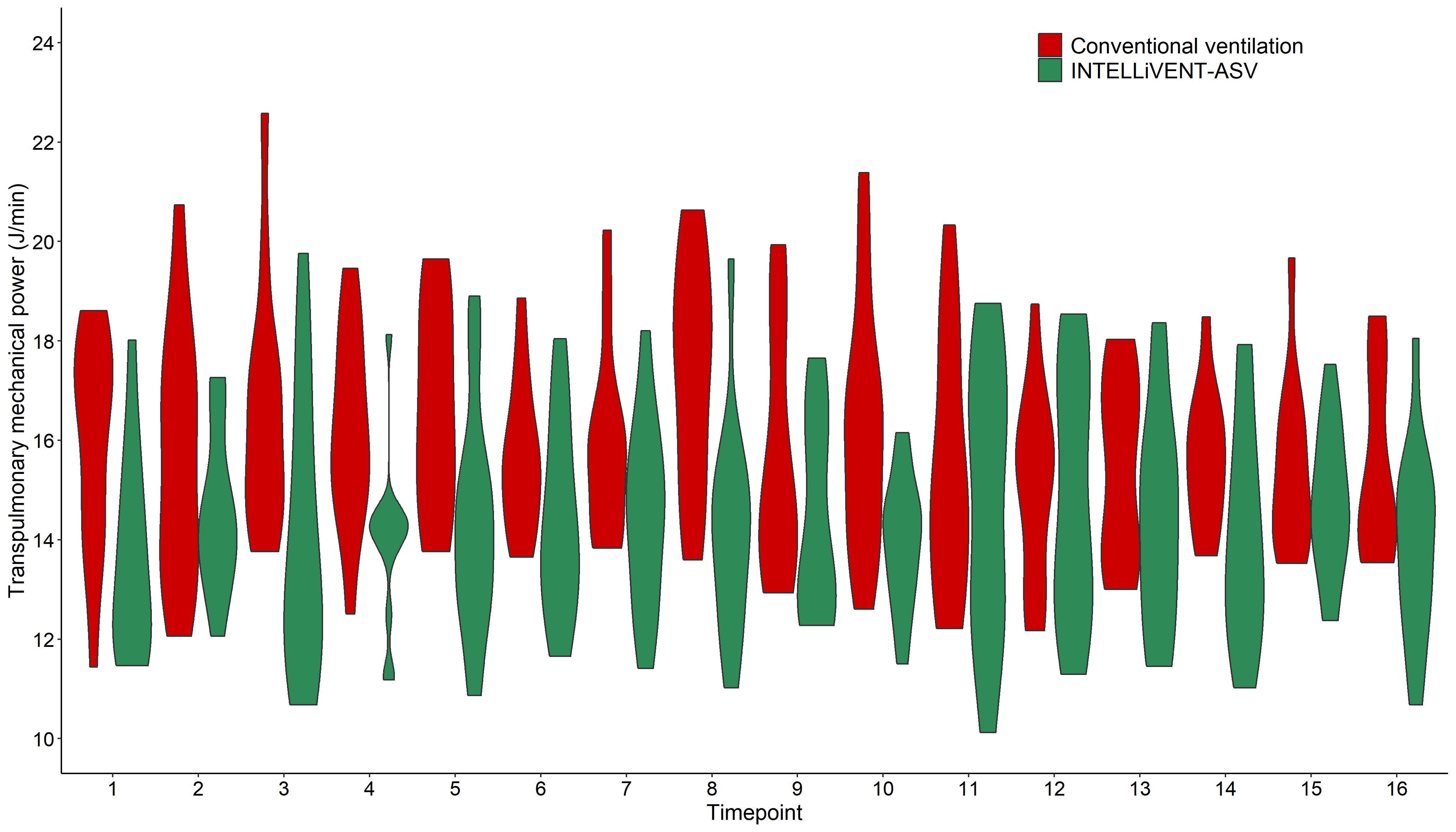
**

**Figure S13.** Violin plot of pairwise comparisons at individual time points of transpulmonary MP.
